# Untargeted Phenolic Profiling and Functional Insights of the Aerial Parts and Bulbs of *Drimia maritima* (L.) Stearn

**DOI:** 10.3390/plants11050600

**Published:** 2022-02-23

**Authors:** Leilei Zhang, Gokhan Zengin, Mohamad Fawzi Mahomoodally, Evren Yıldıztugay, Sharmeen Jugreet, Jesus Simal-Gandara, Youssef Rouphael, Antonio Pannico, Luigi Lucini

**Affiliations:** 1 Department for Sustainable Food Process, Università Cattolica del Sacro Cuore, Via Emilia Parmense 84, 29122 Piacenza, Italy; leilei.zhang@unicatt.it (L.Z.); luigi.lucini@unicatt.it (L.L.); 2 Department of Biology, Science Faculty, Selcuk University, Konya 42130, Turkey; 3 Department of Health Sciences, Faculty of Medicine and Health Sciences, University of Mauritius, Réduit 80837, Mauritius; f.mahomoodally@uom.ac.mu (M.F.M.); sharmeenjugs@gmail.com (S.J.); 4 Deparment of Biotechnology, Science Faculty, Selcuk University, Konya 42130, Turkey; eytugay@gmail.com; 5 Nutrition and Bromatology Group, Department of Analytical Chemistry and Food Science, Faculty of Science, Universidade de Vigo, E-32004 Ourense, Spain; jsimal@uvigo.es; 6 Department of Agriculture, University of Naples Federico II, 80138 Naples, Italy; youssef.rouphael@unina.it

**Keywords:** metabolomics, antioxidants, enzyme inhibition, multivariate analysis, polyphenols

## Abstract

*Drimia maritima* (L.) Stearn (squill), belonging to the Asparagaceae family, is acknowledged as a medicinally valuable species from the *Drimia* genera. In this study, water, methanol, and ethyl acetate extracts of *D. maritima* aerial parts and bulbs were investigated for their polyphenols profile and evaluated for their antioxidant and enzyme inhibition properties. Phenolics were profiled through an untargeted metabolomics approach using an ultra-high pressure liquid chromatograph coupled to quadrupole-time-of-flight mass spectrometry (UHPLC-QTOF-MS). This analysis revealed an enrichment of low molecular weight phenolics and flavonoids in the aerial parts of *D. maritima*, while lignans mainly characterized bulb extracts. Antioxidant capacity was investigated by different assays, including phosphomolybdenum assays, radical scavenging (DPPH: 2,2-diphenyl-1-picrylhydrazyl; ABTS: 2,2′-azino-bis(3-ethylbenzothiazoline-6-sulfonic acid)), as well as reducing ability (CUPRAC: cupric reducing antioxidant capacity; FRAP: ferric reducing antioxidant power), and metal chelating. In radical scavenging and reducing power assays, the water extract of aerial parts exhibited the strongest ability (DPPH: 36.99 mg trolox equivalent (TE)/g; ABTS: 85.96 mg TE/g; CUPRAC: 87.37 mg TE/g; FRAP: 55.43 mg TE/g). In general, the ethyl acetate extracts from aerial parts and bulbs provided the weakest antioxidant capacity. Concerning enzyme inhibitory activities, the water extracts of the bulb were poorly active, while the ethyl acetate extracts from both plant portions displayed the best α-amylase inhibitory abilities. The best acetylcholinesterase (AChE) and butyrylcholinesterase (BChE) abilities were recorded by ethyl acetate extract of aerial parts (2.36 mg galantamine equivalent (GALAE)/g) and bulbs (5.10 mg GALAE/g), respectively. Overall, these results support the medicinal aptitude of *D. maritima* and its possible use as a natural source of antioxidants and enzyme inhibitors with functional potential.

## 1. Introduction

Medicinal plants have been traditionally exploited as a therapeutic resource for local communities worldwide, currently remaining as a primary healthcare model for approximately 85% of the world’s population, and serving as sources for drug discovery [1]. Over the years, many plants have been documented and studied for their potential medicinal attributes, unveiling promising pharmacological properties to manage and treat a wide range of diseases [2,3,4,5].

*Drimia* can be included among the well-described genera consisting of medicinally important species. This genus (Family: Asparagaceae) is comprised of 99 accepted species. The members of this genus are mostly deciduous and rarely evergreen with an underground bulb [6]. Ethnomedicinal applications of numerous *Drimia* species have been widely reported, including the treatment of gout, bronchitis, asthma, and the use as expectorant and emetic agents [7,8]. To date, many studies have been conducted to identify and discover the chemical constituents and characterize the pharmacological properties of *Drimia* species, also exploring different organ parts [9,10,11,12,13]. Indeed, the bulbs accumulate its major bioactive principles, such as cardiac glycosides, phenolic compounds, phytosterols, and other phytochemicals [14]. Nevertheless, the scientific community has also considered the toxicological aspects of *Drimia* species [15,16,17].

*Drimia maritima* (L.) Stearn (synonym: *Urginea maritima* (L.) Baker), commonly known as squill, is native to the Mediterranean region, Africa, and India [18]. Modern clinical studies confirmed the traditional uses of squill in treating non-alcoholic fatty liver, asthma, head lice, alopecia, and inflammatory pains [19]. Additionally, biological properties such as the antioxidant, antibacterial, anticancer, and insecticidal effects of squill have significantly been evidenced in several experimental studies [20,21,22,23,24]. Furthermore, the results from a pilot, triple-blind, randomized clinical trial showed preliminary evidence for the safety and efficacy of the add-on treatment of Squill Oxymel (a traditional formulation from *D. maritima*) in asthmatic patients [18].

Hence, given the remarkable medicinal virtues attributed to *Drimia* species and *D. maritima* in particular, novel approaches are required to add value to this medicinal plant. Thus, the present study is aimed at investigating the efficiency of different solvents in extracting phenolic compounds from different organs and provide insight into *Drimia* antioxidant and enzyme inhibitory capacity. For this purpose, the untargeted phenolic profiling of *D. maritima* extracts was performed together with the determination of in vitro bioactivities. This information will contribute to the existing scientific knowledge about the pharmacological aspects of this species, allowing its exploitation by pharmaceutical, food, and agriculture industries. 

## 2. Results

### 2.1. Untargeted Screening of Polyphenols Content in the Bulb and Aerial Parts of D. maritima

In the present study, three types of solvents, namely ethyl acetate (EA), methanol (MeOH), and water (H_2_O), were used to extract bioactive compounds from aerial parts and bulbs of *D. maritima*. Firstly, these extracts were investigated spectrophotometrically in terms of total phenolic content (TPC) and total flavonoid content (TFC), as shown in the Supplementary Table S1. The aerial parts of *D. maritima* extracts showed TPC ranging from 19.99–31.56 mg gallic acid equivalent (GAE)/g and TFC ranging from 10.15–34.67 mg rutin equivalent (RE)/g. However, the bulb extracts were observed to possess lower TPC (10.39–20.27 mg GAE/g) and TFC (1.06–4.25 mg RE/g). Considering the different solvents employed in the extraction process, water and methanol had a statistically higher extraction capacity for total phenolics and total flavonoid compounds, respectively, in the aerial parts (*p* < 0.05). However, these solvents were not optimal when applied to the bulbs, where EA showed better recovery of the bioactive compounds (Supplementary Table S1).

Afterwards, the phenolic profiles were comprehensively investigated using an untargeted metabolomic approach based on ultra-high pressure liquid chromatograph coupled to quadrupole-time-of-flight mass spectrometry (UHPLC-QTOF-MS). This approach allowed to putatively annotate 471 phenolic compounds, primarily characterized by 255 flavonoids (72 flavonols, 67 anthocyanins, 46 flavones, and 70 other flavonoids), followed by 29 lignans, 80 low molecular weight phenolics, 97 phenolic acids, and 10 stilbenes. The comprehensive list of phenolic compounds annotated, with individual abundances, is reported as supplementary material (Supplementary Table S2). As expected, this approach allowed annotating characteristic polyphenols for each portion of *D. maritima*. Notably, the aerial part extracts were mainly enriched in anthocyanins, namely pelargonidin 3-*O*-, cyanidin 3-*O*-, peonidin 3-*O*-, and petunidin 3-*O*- glycoside, and flavones (apigenin mono- and di- glycosides, isorhoifolin, rhoifolin, chrysoeriol 7-*O*-apiosyl-glucoside, and luteolin 7-*O*-glycoside). The bulb extracts were particularly enriched in lignans characterized mainly by matairesinol derivatives (7-hydroxymatairesinol, 7-oxomatairesinol, and dimethylmatairesinol), followed by sesamolinol, pinoresinol, and conidendrin. In addition to lignans, two flavones with significant abundances were also found, namely sinensetin (or tangeretin) and tetramethylscutellarein (see Supplementary material).

**Figure 1:**
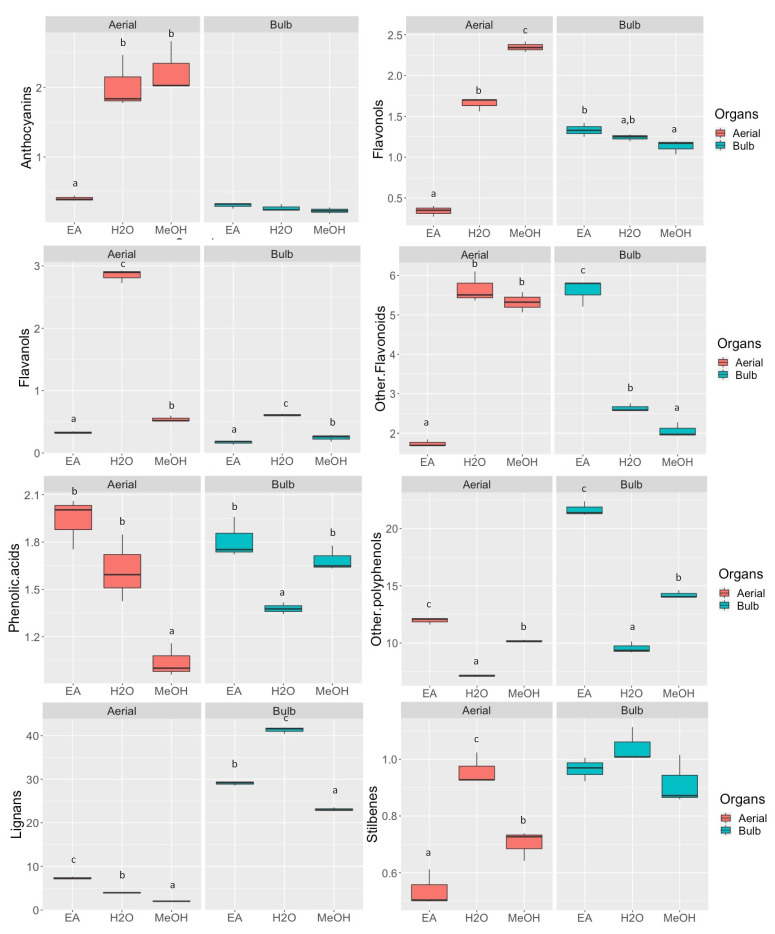
The semi-quantitative result represented in box plots describing the distributions (*n* = 3) of the three different extraction solvents used, i.e., ethyl acetate (EA), water (H_2_O), and methanol (MeOH), in relation to the quantity of bioactive compounds found (expressed as mg eq./g), both for aerial (red box) and for bulbs (green box) parts of *D. maritima*. The letters above each box indicate homogeneous subclasses resulting from ANOVA (*p* < 0.05; Duncan’s post hoc test).

To investigate the effect of the extraction solvents on the recovery of the different classes of bioactive compounds, semi-quantitative analysis was performed using the entire dataset obtained from UHPLC-QTOF-MS analysis. Therefore, we proceeded to classify all the compounds by ontologies and then quantified using standard curves expressing the results as mg equivalent/g of dry extract. The results of the semi-quantitative analysis were reported in Supplementary Table S3. The semi-quantitative data of the different extraction solvents used were represented in box-plot graphs divided by aerial and bulb parts, for easy reading. Box-plot charts were also created for each class of bioactive compounds (Figure 1).

According to the results obtained from spectrometric measurements, the flavonoids such as anthocyanins, flavonols, flavanols, and other flavonoids of the aerial parts were extracted up to 3-fold more in water and methanol (*p* < 0.05), compared to ethyl acetate. On the contrary, phenolic acids, lignans, and other polyphenols were significantly more soluble in ethyl acetate (Figure 1). On the other hand, *D. maritima* bulb extracts have been shown to have a lower flavonoids content compared to the aerial parts, particularly for anthocyanins. In the case of other polyphenols and lignans, which have been shown to have from 2- to 4-fold higher amounts in bulb extracts, the choice of extraction solvent was critical for an enhanced recovery of bioactives. Specifically, other polyphenols characterized by small molecular weight phenolics were efficiently extracted in ethyl acetate and methanol.

### 2.2. Effect of the Extraction Solvents on the Phenolic Profile of D. maritima Organ Parts

**Figure 2:**
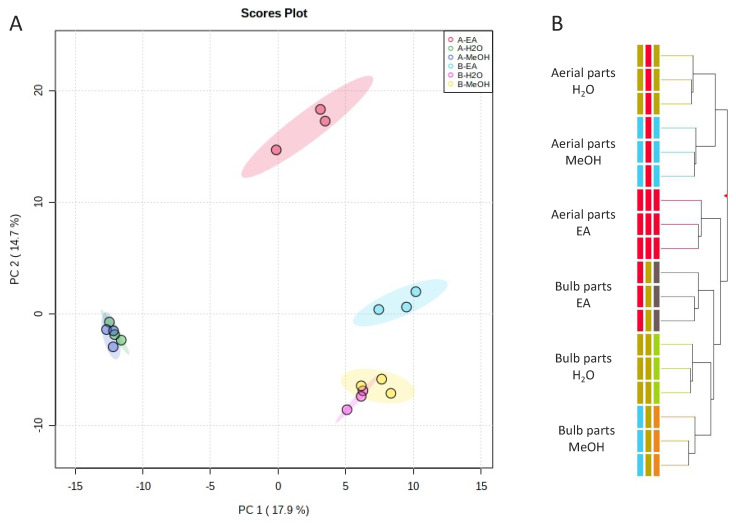
(**A**) Principal component analysis (PCA) and (**B**) hierarchical cluster analysis (HCA) of the aerial and bulbs parts of *D. maritima* extracted with three solvents, i.e., ethyl acetate (EA), water (H_2_O), and methanol (MeOH). In the PCA, the first two axes accounted for 32.6% of sample’s variance. Observations that can be associated with a variable group were delineated with colored cycles.

To investigate the effect of different extraction solvents on the phenolic profile of bulbs and aerial parts of *D. maritima*, multivariate statistics analysis was applied to the metabolomics data. In this regard, unsupervised principal component analysis (PCA) and hierarchical cluster analysis (HCA) were carried out to group samples according to their phytochemical profiles (Figure 2). As reported in Figure 2A, the two principal components of PCA reported 32.6% of the total sample’s variance, defining three clear variable groups: (1) aerial parts extracted with methanol and water, (2) aerial parts extracted with ethyl acetate, and (3) bulb extracts obtained by using the three extraction solvents. This samples grouping was also confirmed with HCA (Figure 2B). Overall, these models confirmed a large diversity in the phenolic profile of the aerial and bulb parts of *D. maritima*. Furthermore, the models suggest that, in addition to the differences due to organ parts, the three solvents also used could affect the phenolic profiles. This evidence was clear when EA solvent was considered, showing an effective phenolic profile diversity in the case of aerial part extracts and a slight difference in the case of bulb extracts.

Afterwards, a supervised orthogonal projection to latent structures discriminant analysis (OPLS-DA) was built considering different extraction solvents for both aerial (Figure S1A) and bulb (Figure S1B) organ parts to better separate samples according to their phenolic profile. Interestingly, a complete separation among three solvents was achieved by two latent vectors for both models, claiming a distinctive phytochemical profile among them. The OPLS-DA score plot models were characterized by excellent model parameters of R^2^Y (the goodness-of-fit) = 0.99 and Q^2^Y (goodness-of-prediction) = 0.95. To identify the most discriminant compounds contributing to the differences outlined in the OPLS-DA, the variable importance in projection (VIP) compounds was selected with a VIP score ≥ 1.2 and statistically significant (*p* < 0.05) (Supplementary Table S4). As reported, the most discriminant compounds for the aerial parts of *D. maritima* extracts were mainly driven by flavonoids (pelargonidin, apigenin, and isorhamnetin 3-*O*-glucuronide), followed by phenolic acids and other polyphenols (tyrosols and 5-heneicosylresorcinol). Similarly, for bulb extracts, the VIP markers were mainly characterized by the flavonoids theaflavin, luteolin 6-*C*-glucoside, hihydroquercetin, and catechin 3-*O*-glucose, being efficiently extracted in methanolic and ethyl acetate solvents. However, most classes of phenolic compounds such as lignans, low molecular weight phenolics, and some flavonoids were shown effectively extracted in water and ethyl acetate solvents as secoisolariciresinol-sesquilignan, medioresinol, 5-heptadecylresorcinol, and quercetin 3-*O*-glucosyl-rhamnosyl-galactoside.

### 2.3. In Vitro Antioxidant Activities

In the current study, the *D. maritima* extracts were tested for their antioxidant potential as radical scavenging, reducing, metal chelating agents. All the extracts showed radical scavenging ability in 2,2-diphenyl-1-picrylhydrazyl (DPPH: 4.75–36.99 mg trolox equivalent (TE)/g) and 2,2′-azino-bis(3-ethylbenzothiazoline-6-sulfonic acid (ABTS: 23.51–85.96 mg TE/g) assays (Table 1). The water and ethyl acetate extracts from *D. maritima* aerial and bulb parts, respectively, showed the highest radical scavenger capacity compared to other solvents. This same trend could be observed for the reducing activity of the extracts in cupric reducing antioxidant capacity (CUPRAC) and ferric reducing antioxidant power (FRAP) assays. Indeed, the aerial part water extract and the bulbs ethyl acetate extract showed the most potent activity. In the CUPRAC assay, the reducing activity ranged from 47.44–87.37 mg TE/g for the aerial part extracts and 24.31–66.92 mg TE/g for the bulb extracts. On the other hand, for FRAP assay, the extracts ranged from 15.26–55.43 mg TE/g (Table 1). 

Metal chelating activity was also detected in all extracts (aerial part: 21.18–25.28 mg EDTA equivalent (EDTAE)/g; bulbs: 3.78–13.21 mg EDTAE/g) (Table 1). Total antioxidant capacity has been confirmed as a better way to depict the combined effect of phenolics, flavonoids, and other reducing compounds in the plant extracts [25]. Phosphomolybdenum assay revealed that all extracts possess the total antioxidant capacity (0.72–1.45 mmol TE/g), reporting the EA extracts with the highest activity (Table 1).

### 2.4. Inhibitory Capacity on Key Enzymes

To detect enzyme inhibitory effects of the tested extracts, cholinesterases (AChE and BchE), tyrosinase, α-amylase, and α-glucosidase were selected as target enzymes. The results are given in Table 2. In the present study, except for water extracts, all the extracts reported the ability to inhibit AChE (0.36–2.36 mg GAE/g) and BChE (1.65–5.10 mg galantamine equivalent GALAE/g; Table 2). The best AChE and BChE inhibitory ability was found in ethyl acetate extract of aerial parts and bulb, respectively. Water extracts had lower cholinesterase inhibitory abilities compared to methanol and water extracts. 

In the present study, all extracts except the bulbs water extract showed tyrosinase inhibitory potential (6.44–54.64 mg kojic acid equivalent (KAE)/g). Although the aerial part water extract showed a slight inhibitory capacity against tyrosinase (Table 2). The best tyrosinase inhibitory activity was found in the methanol extract of aerial parts. 

Interestingly, all extracts inhibited both amylase (0.17–1.00 mmol acarbose equivalent (ACAE)/g) and glucosidase (0.04–0.66 mmol ACAE/g), except the methanolic and water bulb extracts of *D. maritima*, which exclusively inhibited amylase (0.53 and 0.09 mmol ACAE/g). However, the ethyl acetate extracts possessed better inhibitory properties against glycosidase enzymes. In general, the bulbs water extract was a poor inhibitor of amylase and glucosidase (Table 2). 

### 2.5. Pearson’s Correlation between Phenolic Profiles and Biological Activities

**Figure 3:**
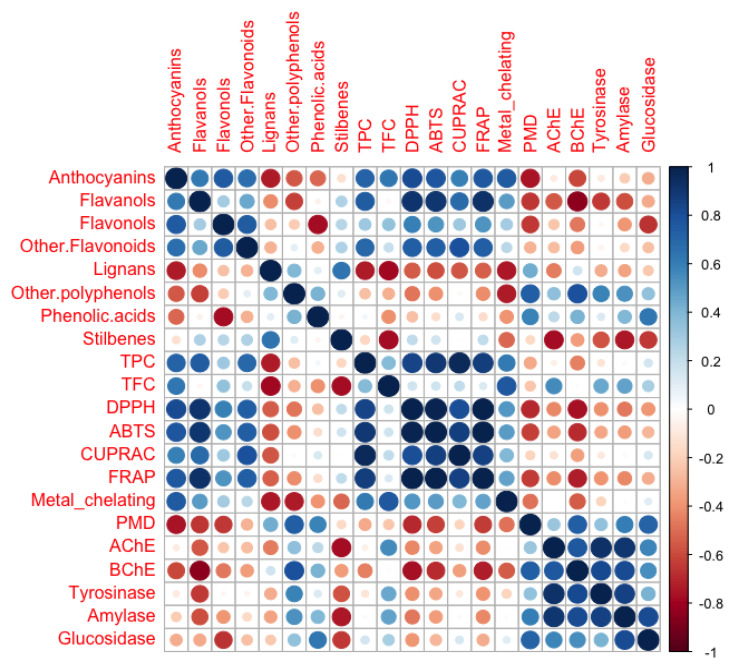
Correlogram of different classes of polyphenols and biological activities. Colored cycles indicate different degree of correlation coefficient (r), ranging from dark blue as r = 1 (positive correlation) to dark red as r = −1 (negative correlation). Abbreviations: TPC: Total Phenolics Content; TFC: Total Flavonoids Content; PMD: Phosphomolybdenum; AChE: acetylcholinesterase; and BchE: butyrylcholinesterase.

The Pearson’s correlation coefficient (*r*) between different classes of polyphenols and biological activities were calculated to identify a possible relationship among them. Individual Pearson’s correlation coefficients and *p*-values are provided in the supplementary material (Table S5). As shown in the correlogram (Figure 3), a relevant correlation was observed between flavonoids such as anthocyanins, flavanols, and other flavonoids with antioxidant assays. in particular, the best correlation was found between flavanols and DPPH, ABTS, and FRAP assays (showing correlation coefficients higher than 0.9, *p* < 0.01; supplementary material). It is interesting to note that monomeric catechins and dimeric/trimeric procyanidins were the most abundant flavanols in the *D. maritima* extracts.

On the other hand, other polyphenols and phenolic acids classes were reported to be linearly correlated with enzyme inhibitory capacity, i.e., AChE, BChE, tyrosinase, α-amylase, and α-glucosidase activities (Figure 3). Specifically, low molecular weight phenolics, characterized mainly by phenolic diterpenes as carnosol, carnosic acid, rosmadial, and carvacrol, were shown to be highly correlated with the inhibition capacity of BChE (*r* = 0.79; *p* < 0.01) and moderately correlated with inhibition capacity of α-amylase, and α-glucosidase (*r* = 0.53–0.58; *p* < 0.05). Moreover, phenolic acids were detected having a modest relationship with α-glucosidase inhibition capacity. On the contrary, lignans and stilbenes showed a generally negative correlation with the biological activities tested, suggesting a lack of contribution from these classes on the antioxidants and enzymes inhibition capacity of *D. maritima*.

## 3. Discussion

### 3.1. Untargeted Screening of Polyphenols Content in the D. maritima Extracts

The scientific investigation of plants recognized for their medicinal value is a valuable strategy to discover novel therapeutic agents [26]. For instance, phenols and flavonoids are the most common phytoconstituents of different fruits, vegetables, and medicinal and aromatic plants, responsible for antioxidant activities. Plants contain polyphenols that act as free radical scavengers and reduce oxidative stress and could be employed as a remedy to cure various harmful human ailments [27]. The potential applications of these phytochemicals from medicinal plants, in pharmaceutical and medical aspects, especially for antioxidant, cardioprotective, antibacterial, anticancer, immune system promoting, and anti-inflammatory effects, have been extensively highlighted by [28]. Notably, the objective of the extraction process is to maximize the amount of target compounds and obtain the highest biological activity from plant extracts. The extraction yield and biological activity of the resulting extract are not only affected by the extraction methodology, but also by the extraction solvent used. Many solvents, including methanol, ethanol, water, and acetone, have been used for extracting bioactive compounds from plant materials [29]. In the present study, three types of solvents, i.e., ethyl acetate, methanol, and water were used to extract the total bioactive compounds in the two organ parts of *D. maritima* (aerial and bulbs).

Overall, *D. maritima* aerial parts extracts showed higher flavonoid content compared to bulbs, particularly for anthocyanins and flavones, which were best extracted in water and methanol solvents. The results were in agreement with Vega and Martin [30], who found cyanidin-3-monoglucoside as the most abundant anthocyanin in the aerial parts of *Urginea maritima* Baker. In accordance to the literature, the OPLS-DA model of the *D. maritima* aerial parts reported classes of flavonoid and anthocyanin compounds (e.g., pelargonidin, apigenin, and isorhamnetin 3-*O*-glucuronide), to be the best discriminants in *D. maritima* extracts [22,31]. The bulb extracts resulted rich in low molecular weight phenolic compounds and lignans, e.g., secoisolariciresinol-sesquilignan, medioresinol, 5-heptadecylresorcinol, and quercetin 3-*O*-glucosyl-rhamnosyl-galactoside. These classes of compounds were efficiently extracted in ethyl acetate and methanol. The high diversity of polyphenols in the *D. maritima* extracts confirms and extends previous literature, reporting a broad profile including different compounds classes. Rhimi et al. [32] characterized the chemical profile of *Drimia maritima* bulb and *Dittrichia viscosa* leaf extracts and identified 29 compounds including quercetin, kaempferol, and bufadienolides as the major components. The same characterization was performed by Kakouri et al. [31], which reported that aerial parts of *Drimia numidica* exhibited the highest phenolic content. Authors identified phenolic acids and flavonoids such as chlorogenic acid, *p*-coumaroyl quinic acid, apigenin pentoside-hexoside, and kaempferol hexoside.

### 3.2. Antioxidant Activities

Over the years, research on antioxidants as potential therapeutic agents to prevent free radical-generated damage in the human body, has gained enormous popularity. Natural antioxidants have attracted considerable attention by consumers and researchers over their synthetic counterparts [33]. Antioxidants present multiple mechanisms of action, ranging from free-radicals scavenging to metal ion chelation, among others [34]. Consequently, several complementary assays were used for assessing the antioxidant activity of *D. maritima* extracts. All *D. maritima* extracts were reported to exert radical scavenging ability, as reported by DPPH and ABTS assays, as well as reducing potential activity, by means of CUPRAC and FRAP assays. Previous studies have reported the antioxidant potential of the plant *D. maritima* [20,35]. For instance, in the study of Belhaddad et al. [24], the DPPH method showed that the methanolic extract of *U. maritima* has a free-radical scavenger effect with an inhibitory concentration 50 (IC_50_) of 57.83 ± 1.59 µg/mL. The same study reported that the fractions isolated from *U. maritima* had an IC_50_ ranging between 499.23 and 39.68 µg/mL. In another study, the antioxidant properties of D. *maritima* ethyl acetate flower extracts showed the highest reducing power and scavenging activity against DPPH. These authors also found that the aqueous extract was the most effective for the scavenging of hydroxyl radical, superoxide anion, as well as inhibiting lipid peroxidation. Phenolic determination revealed that D. *maritima* flowers contain phenolic compounds, flavonoids, and tannins [36]. Other studies have also shown a strong correlation between the phenolic content and the antioxidant activities of extracts [25,37]. Accordingly, the best correlation coefficient (0.9 and *p* < 0.01; supplementary material) was found between flavanols and DPPH, ABTS, and FRAP assays. It is interesting to note that the most abundant flavanols in the *D. maritima* extracts were monomeric catechins and dimeric/trimeric procyanidins. The antioxidant capacity of catechin and procyanidin was also confirmed by TBARS and TEAC methods [38], and were evaluated for their application as suitable sources for the preparation of flavanol-rich antioxidant extracts. Moreover, procyanidins extracted and concentrated from *Vaccinium* wild berries exhibited a significant DPPH scavenging activity [39]. Additionally, other *Drimia* species, i.e.,: *D. coromandeliana*, *D. govindappae*, *D. indica*, *D. nagarjunae*, *D. polyantha*, *D. raogibikei*, and *D. razii* were studied on the basis of phenolic content and antioxidant activity, showing that *D. coromandeliana* contains the highest phenolic content the strongest free radicals scavenging activity [10].

### 3.3. Enzyme Inhibition Activities

#### 3.3.1. Cholinesterase Inhibition Capacity

Cholinesterase is a family of enzymes that essentially contains acetylcholinesterase (AChE) and butyrylcholinesterase (BChE). Ethno-pharmacological approaches and bioassay-guided isolation have provided a way to find potential AChE and BChE inhibitors from plants [40]. Consequently, over recent years, a large number of such inhibitors have been isolated from medicinal plants [41,42]. Among them, *D. maritima* extracts developed the ability to inhibit both enzymes, with the exception of water extracts, thus confirming previous results [22]. The correlation analysis reported a direct relationship between LMW and other polyphenols and AChE and BChE enzyme inhibitory capacity. Specifically, LMW phenolics, characterized mainly by phenolic diterpenes as carnosol, carnosic acid, rosmadial, and carvacrol, were shown to be highly correlated with the inhibition capacity of BChE (*r* = 0.79; *p* < 0.01). Nowadays, many studies reported the neurobiological activities of phenolic diterpenes. Specifically, carnosic acid has been shown to develop a positive effect on the Alzheimer’s disease (AD), by preventing the formation of senile plaques associated with this ailment [43]. Moreover, carnosic acid and carnosol extracted from *Lepechinia mutica* showed a higher anti-BChE activity than that of synthetic drugs such as Donepezil [44]. Furthermore, Topcu et al. [45] reported the efficacy of seven diterpenoids extracted from *Salvia fruticosa*, including carnosic acid and derivatives against BChE, resulting in a new target to manage AD [43].

#### 3.3.2. Tyrosinase Inhibition Capacity

Tyrosinase inhibitors are among the most promising solutions to counter the eventual undesirable effects associated with melanogenesis [46], with a large demand in the cosmetic and medicinal industry due to their preventive effect on pigmentation disorders and skin-whitening effect [47]. Indeed, existing evidence [48] suggests that some phytochemicals present tyrosinase and melanogenesis inhibitor properties. In the present study, either methanolic or ethyl acetate extracts from *D. maritima* seemed to be effective tyrosinase inhibitors, likely due to the accumulation of phenolic compounds. The anti-tyrosinase effect of squill has also been reported in an earlier study. For instance, the inhibitory activities of the *U. maritima*, *Zhumeria majdae,* and *Physalis divaricata* against mushroom tyrosinase were 38.61, 29.70, and 25.74% at 1.67 mg/mL, respectively [49]. In parallel, the ethanolic extract from *Asphodelus microcarpus* flowers showed the strongest inhibitory effect on tyrosinase activity, accompanied by the highest antioxidant activity and elevated levels of total phenolics and flavonoids [50]. However, such a relationship was not observed in the present study, where the aerial part water extract showed the highest antioxidant potential and a weak anti-tyrosinase activity, compared to the other extracts.

#### 3.3.3. α-Amylase and α-Glucosidase Inhibition Capacity

Novel approaches are required to prevent the incidence of prevalent diseases, including diabetes. In this sense, the inhibition of glycolytic enzymes, such as α-amylase and α-glucosidase, constitutes a molecular-based approach to that aim [51]. There is plenty evidence about the antidiabetic properties of phenolic compounds from medicinal plants, based on their ability to increase insulin secretions or slowing the intestinal absorption of glucose [52,53]. Furthermore, the proven safety of phenolic compounds may represent a beneficial solution to counter the side effects attributed to synthetic drugs such as diarrhea and other intestinal disturbances [54]. As a result, in the search for alternative antidiabetic agents, a large number of plants has been screened for potential hypoglycaemic activity [55,56,57,58]. 

Interestingly, the methanolic and water bulb extracts of *D. maritima* selectively inhibited α-amylase, as previously reported [34]. Results indicated that the extract exhibited a considerable inhibitory effect at all tested concentrations with an IC_50_ of 95.03 ± 1.29 µg/mL. Additionally, the anti-α-amylase activity was significantly correlated to the profile of *U. maritima* bulbs extracts. In turn, the ethyl acetate bulb extracts inhibited both α-amylase and α-glucosidase, the latter exhibiting a stronger inhibition. Other researchers have investigated the carbohydrate digestive inhibitory activity of other *Drimia* species. Notably, among all the tested extracts of *D. nagarjunae*, Alluri and Majumdar [59] reported that leaves and bulbs aqueous extracts exhibited the inhibitory activity against α-glucosidase in a dose-dependent manner.

According to the literature, a modest correlation between the phenolic acids and other polyphenols and α-amylase and α-glucosidase inhibition capacity was detected. Generally, Tan et al. [60] reported a linear correlation among phenolic content, antioxidant capacity, and inhibition of α-amylase and α-glucosidase. According to this research, a direct involvement of phenolic acids in the modulation of starch digestive enzymes activities was postulated by other authors [61,62]. In this regard, the effectiveness of phenolic acids as inhibitors of carbohydrate hydrolyzing enzymes has been related to the presence of a unique hydroxyl group on their structure [63].

## 4. Materials and Methods

### 4.1. Plant Material

*Drimia maritima* samples were collected in the city of Mersin (between Gülnar and Aydıncık, Turkey) in the 2020 summer season. The plants were confirmed by one of the botanist co-author (Dr. Evren Yıldıztugay), Konya, Turkey, and one voucher specimen (No: EY-3070) has been deposited in Selcuk University. The aerial parts and bulbs of the plant samples were dried in shade conditions for 10 days at room temperature. The plant samples were powdered using a laboratory mill, and the powdered plant samples were stored in dark conditions at room temperature.

### 4.2. Extraction Methods

In the present work, we used ethyl acetate, methanol, and water as solvents. Maceration was used as an extraction method, together with infusion in water. The plant materials (10 g) were mixed with 200 mL solvents (ethyl acetate or methanol) at room temperature for 24 h. Then, all extracts were filtered, and the solvents were removed using a rotary evaporator. Regarding water extracts, the plant materials (10 g) were infused with 200 mL boiling water for 15 min and then filtered. Water extracts were lyophilized, and all extracts were stored at 4 °C until analysis.

### 4.3. Determination of Total Bioactive Compounds, In Vitro Antioxidant and Enzyme Inhibitory Activities

The TPC and TFC were determined according to previously described methods [64,65]. TPC was expressed as mg GAE/g dry extract, whereas TFC was expressed as mg RE/g dry extract. In the current work, the antioxidant effects of the tested extracts were detected by different assays [64]. The assays were [1,1-diphenyl-2-picrylhydrazyl (DPPH) and 2,2′-azino-bis(3-ethylbenzothiazoline) 6-sulfonic acid (ABTS) radical scavenging, cupric ion reducing antioxidant capacity (CUPRAC), ferric ion reducing antioxidant power (FRAP), metal chelating ability (MCA), and phosphomolybdenum assay (PDA)]. For DPPH, ABTS, CUPRAC, and FRAP assays, data were expressed as mg Trolox equivalents (TE)/g extract, whereas in MCA and PDA, mg EDTA equivalents (EDTAE)/g extract and mmol TE/g extract, respectively, were used. The experimental parts for acetylcholinesterase, butyrylcholinesterase, tyrosinase, α-amylase, and α-glucosidase assays were previously provided. Galanthamine was used as a positive control in cholinesterase assays, and data were evaluated as mg GALAE/g extract. Kojic acid was used as a standard inhibitor in tyrosinase inhibitory assay, and the results were expressed as mg KAE/g extract [64,65]. Acarbose was selected as inhibitors for α-amylase and α-glucosidase inhibitory assays, and the results are given as mmol ACAE/g extract.

### 4.4. Metabolomics Analysis

The untargeted metabolomics analysis was carried out using 0.5 g dried samples of *D. maritima* extracts and solubilized in 5 mL of aqueous methanol with 0.1% formic acid (Merck KGaA, Darmstadt, Germany). The solubilized samples were further centrifuged at 8000× *g* for 15 min, filtered with a 0.22 mm syringe filter, and transferred in glass vials. The phenolic profiles of different *D. maritima* extracts were analyzed using a UHPLC-QTOF-MS (Agilent Technologies, Stevens Creek Blvd, Santa Clara, CA, USA). The instrument parameters and methods adopted for the analysis were optimized in previous works [66,67]. Regarding chromatographic separation, it was achieved by using an Agilent InfinityLab Poroshell 120 pentafluorophenyl (PFP) column (2.1 × 100 mm, 1.9 μm) (Agilent Technologies, Stevens Creek Blvd, Santa Clara, CA, USA) and a binary mixture of water and acetonitrile acidified with 0.1% (*v*/*v*) formic acid as mobile phase (LC-MS grade, VWR, Milan, Italy).

The post-acquisition data filter was carried out using Agilent Profinder B.10.0 (Agilent Technologies, Santa Clara, CA, USA), retaining only those compounds putatively annotated within 75% of replications in at least one condition. The identification of phenolic compounds was carried out according to the ‘find-by-formula’ algorithm, against the Phenol-Explorer database [68,69]. Monoisotopic accurate mass was used considering the entire isotopic profile, achieving the COSMOS level 2 of confidence in annotation [70]. The semi-quantitative analysis of different phenolic classes was performed using phenolic standard curves of cyanidin, catechin, luteolin, ferulic acid, sesamin, resveratrol, and tyrosol (analytical grade, Sigma-Aldrich, S. Louis, MO, USA). Results were expressed as mg phenolic equivalents/g dry extract. The grouped box plots were carried out using R-studio software.

### 4.5. Statistical Analysis

The statistical analysis was performed for each biological assay and semi-quantitative value within each phenolic class, through one-way analysis of the variance (ANOVA) using the software PASW Statistics 25.0 (SPSS Inc., Chicago, IL, USA) (*p* < 0.05, Duncan’s post hoc test). Correlogram, Pearson’s correlation coefficients, and *p*-value matrix analyzed among different phenolic classes and biological activities were performed using R-studio software. 

The raw metabolomic data set was transformed and normalized in Mass Profiler Professional B.15.1 (Agilent Technologies, Stevens Creek Blvd, Santa Clara, CA, USA). Therefore, chemometric interpretation such as Principal component analysis (PCA) was performed by using MetaboAnalyst 5.0 (https://www.metaboanalyst.ca accessed on 10 December 2022), while the orthogonal projection to latent structures discriminant analysis (OPLS-DA) was created using SIMCA 16 software (Umetrics, Malmo, Sweden), as previously described [67]. To validate and to investigate outliers of the OPLS-DA model, the model fitness parameters (goodness of fit: R^2^Y; goodness of prediction: Q^2^Y; cross-validation: CV-ANOVA, *p* < 0.01) were determined, and permutation test (*n* = 100) and Hotelling’s T2 (95% and 99% confidence limit for the suspect and strong outliers, respectively) performed [71]. Then, the variable importance in projection (VIP > 1.2) approach was used to identify discriminant metabolites.

## 5. Conclusions

In this study, different extracts of *D. maritima* were tested for their bioactive contents and pharmacological activities, in terms of their antioxidant and enzyme inhibitory actions. Phenolic profiles differed significantly as a function of the plant organ and extraction solvent, with bulb extracts showing lower flavonoid content compared to the extracts from aerial parts. Water and methanol were found to be relatively better solvents for extracting total phenolics and flavonoids, respectively, from the aerial parts of *D. maritima*. The results also revealed that the water extract of aerial parts provided the highest radical scavenging and reducing potential among all extracts, mostly due to the accumulation of flavanols, especially catechins and procyanidins. Moreover, most of the studied extracts possessed significant inhibitory potential against acetyl- and butyryl- cholinesterase, highly correlated to the presence of phenolic terpenes. Furthermore, extracts also showed inhibitory activity against tyrosinase, as well as α-amylase and α-glucosidase, the latter being correlated to phenolic acids. Overall, these results support the exploitation of *D. maritima* as medicinal plant, considering its possible use as a source of antioxidants and enzyme inhibitory activities. Nonetheless, the correct choice of plant organs and the solvent for extraction are critical steps to support the use of *D. maritima* as a source of functional components.

## Figures and Tables

**Table 1:** Antioxidant properties of the tested extracts. The superscript letters indicate statistically significant comparison from ANOVA (*p* < 0.05; Duncan’s post hoc test) among aerial parts (lower case letters) and bulbs (upper case letters).

Parts	Solvents	DPPH	ABTS	CUPRAC	FRAP	MCA	PBD
		(mg TE/g)				(mg EDTAE/g)	(mmol TE/g)
Aerial parts	EA	4.75 ± 0.40 ^a^	26.52 ± 0.77 ^a^	47.44 ± 0.72 ^a^	15.26 ± 0.75 ^a^	21.18 ± 1.76 ^a^	1.35 ± 0.02 ^c^
	MeOH	19.44 ± 0.31 ^b^	48.34 ± 0.51 ^b^	53.18 ± 0.65 ^b^	29.93 ± 0.46 ^b^	25.28 ± 0.33 ^b^	0.82 ± 0.06 ^b^
	Water	36.99 ± 0.38 ^c^	85.96 ± 1.13 ^c^	87.37 ± 0.16 ^c^	55.43 ± 0.39 ^c^	22.91 ± 0.30 ^a^	0.72 ± 0.04 ^a^
Bulbs	EA	11.44 ± 0.35 ^C^	38.62 ± 0.25 ^C^	66.92 ± 1.69 ^B^	24.49 ± 0.39 ^B^	4.68 ± 0.26 ^B^	1.45 ± 0.09 ^B^
	MeOH	6.81 ± 0.28 ^A^	27.67 ± 0.20 ^B^	25.60 ± 0.27 ^A^	16.52 ± 0.24 ^A^	3.78 ± 0.23 ^A^	0.99 ± 0.05 ^A^
	Water	7.67 ± 0.23 ^B^	23.51 ± 0.62 ^A^	24.31 ± 0.50 ^A^	16.38 ± 0.29 ^A^	13.21 ± 0.40 ^C^	1.09 ± 0.10 ^A^

Values are reported as mean ± SD. EA: Ethyl acetate; MeOH: Methanol; TE: Trolox equivalent; MCA: metal chelating activity; EDTAE: EDTA equivalents; and PBD: Phosphomolybdenum.

**Table 2:** Enzyme inhibitory effects of the tested extracts. The superscript letters indicate statistically significant comparison from ANOVA (*p* < 0.05; Duncan’s post hoc test) among aerial parts (lower case letters) and bulbs (upper case letters).

Parts	Solvents	AChE	BChE	Tyrosinase	Amylase	Glucosidase
		(mg GALAE/g)		(mg KAE/g)	(mmol ACAE/g)	
Aerial parts	EA	2.36 ± 0.09 ^c^	4.77 ± 0.40 ^b^	48.59 ± 0.65 ^b^	1.00 ± 0.01 ^c^	0.66 ± 0.08 ^b^
	MeOH	1.89 ± 0.01 ^b^	2.80 ± 0.24 ^a^	54.64 ± 0.44 ^c^	0.63 ± 0.01 ^b^	0.06 ± 0.02 ^a^
	Water	0.36 ± 0.04 ^a^	na	6.44 ± 0.93 ^a^	0.17 ± 0.01 ^a^	0.04 ± 0.01 ^a^
Bulbs	EA	1.39 ± 0.19 ^A^	5.10 ± 0.27 ^B^	50.18 ± 0.51 ^A^	0.76 ± 0.01 ^C^	0.33 ± 0.05 ^A^
	MeOH	1.86 ± 0.09 ^B^	4.72 ± 0.23 ^B^	51.38 ± 0.57 ^B^	0.53 ± 0.02 ^B^	na
	Water	na	1.65 ± 0.13 ^A^	na	0.09 ± 0.01 ^A^	na

Values are reported as mean ± SD. EA: Ethyl acetate; MeOH: Methanol; GALAE: Galantamine equivalent; KAE: Kojic acid equivalent; ACAE: Acarbose equivalent; and na: not active.

## Data Availability

The metabolomics dataset is provided as supplementary material.

## Supplementary Materials

The following supporting information can be downloaded at: https://www.mdpi.com/article/10.3390/plants11050600/s1, Figure S1: OPLS-DA models; Figure S2: Validation models; Table S1: TPC and TFC; Table S2: Dataset; Table S3: semiquantitative analysis; Table S4: VIP markers; Table S5: Pearson’s correlation.
