# Supplementary material for: Untargeted Phenolic Profiling and Functional Insights of the Aerial Parts and Bulbs of Drimia maritima (L.) Stearn
Source: Plants (Basel). 2022 Feb 23;11(5):600. doi: 10.3390/plants11050600 (PMC8912325; doi:10.3390/plants11050600)
Supplement: Supplementary file 1 [file plants-11-00600-s001.zip › Figure S1 - OPLS-DA models.pdf]

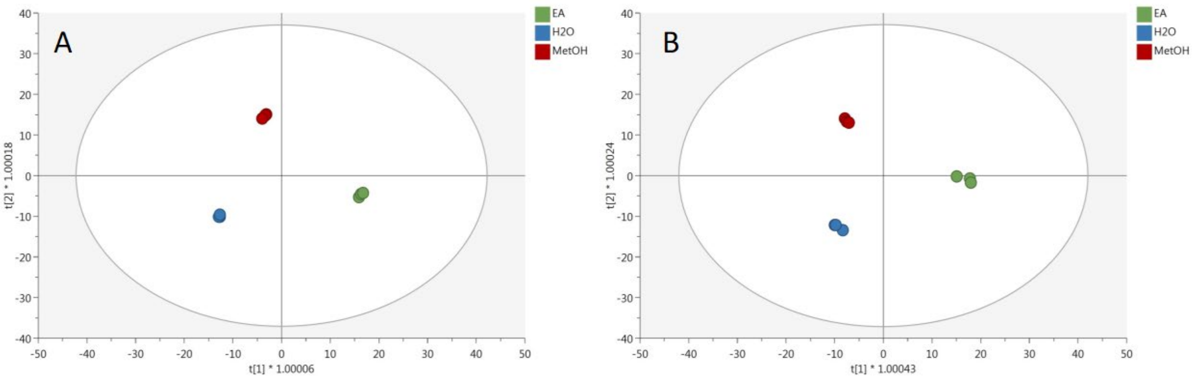

Figure S1. orthogonal projection to latent structures discriminant analysis (OPLS-DA) of the phenolic profile of the different *D. maritima* extracts, considering different extraction solvents *i.e.,* ethyl acetate (EA), water (H2O) and methanol (MetOH) for (A) aerial parts characterized by R^2Y (the goodness-of-fit) = 1 and Q^2Y (goodness-of-prediction) = 0.950, and (B) bulb parts characterized by R^2Y = 1 and Q^2Y = 0.958.
