# Supplementary material for: Untargeted Phenolic Profiling and Functional Insights of the Aerial Parts and Bulbs of Drimia maritima (L.) Stearn
Source: Plants (Basel). 2022 Feb 23;11(5):600. doi: 10.3390/plants11050600 (PMC8912325; doi:10.3390/plants11050600)
Supplement: Supplementary file 1 [file plants-11-00600-s001.zip › Figure S2 - Validation models.pdf]

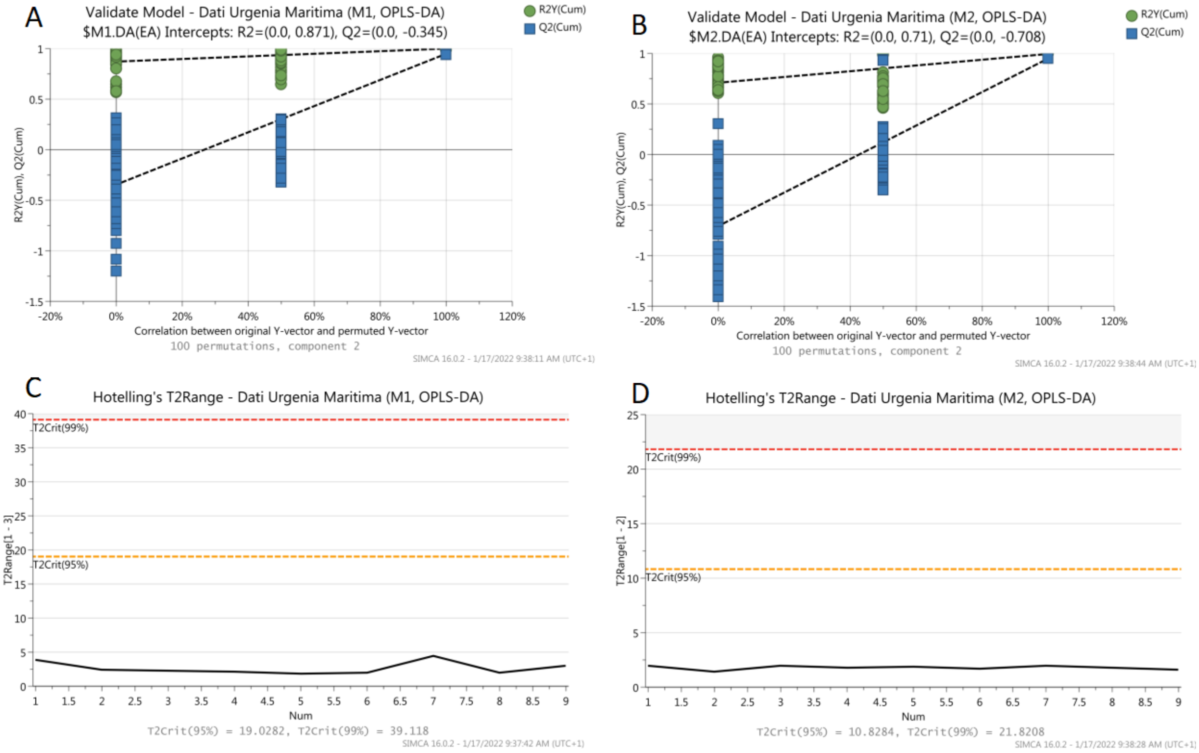


**Figure S2.** Validation of OPLS-DA discriminant model of aerial and bulb *D. maritima* organ parts; Hotelling's T2 using 95% and 99% confidence limits is given in upper pane for aerial parts (A) and bulb parts (B), whereas outcome of permutation test (N = 100) is given in the lower one for aerial parts (C) and bulb parts (D).
