# Supplementary material for: Untargeted Phenolic Profiling and Functional Insights of the Aerial Parts and Bulbs of Drimia maritima (L.) Stearn
Source: Plants (Basel). 2022 Feb 23;11(5):600. doi: 10.3390/plants11050600 (PMC8912325; doi:10.3390/plants11050600)
Supplement: Supplementary file 1 [file plants-11-00600-s001.zip › Table S1 - TPC and TFC.pdf]

Table S1. Total bioactive compounds of the tested extracts.

Parts	Solvents	TPC (mg GAE/g)	TFC (mg RE/g)
	EA	19.99 ± 0.18 [a]	22.32 ± 0.73 [b]
Aerial parts	MeOH	21.58 ± 0.10 [b]	34.67 ± 0.27 [c]
	Water	31.56 ± 0.35 [c]	10.15 ± 0.30 [a]
	EA	20.27 ± 0.15 [B]	4.25 ± 0.09 [C]
Bulbs	MeOH	10.72 ± 0.29 [A]	3.12 ± 0.05 [B]
	Water	10.39 ± 0.02 [A]	1.06 ± 0.16 [A]

[*]Values are reported as mean ±SD. EA: Ethyl acetate; MeOH: Methanol; TPC: Total phenolic content; TFC: Total flavonoid content; GAE: Gallic acid equivalent; RE: Rutin equivalent; TE: Trolox equivalent.
