# Supplementary material for: Untargeted Phenolic Profiling and Functional Insights of the Aerial Parts and Bulbs of Drimia maritima (L.) Stearn
Source: Plants (Basel). 2022 Feb 23;11(5):600. doi: 10.3390/plants11050600 (PMC8912325; doi:10.3390/plants11050600)
Supplement: Supplementary file 1 [file plants-11-00600-s001.zip › Table S3 - semiquantitative analysis.pdf]

Table S3. Semi-quantification per classes of phenolics identified from UHPLC-ESI-QTOF-MS in the different aerial and bulb organ parts of *D. maritima*, considering three different extraction solvents *i.e.*, ethyl acetate (EA), methanol (MeOH), and water.

Parts	Solvents	Anthocyanins	Flavanols	Flavonols	Other Flavonoids	Lignans	Other polyphenols	Phenolic acids	Stilbenes
	EA	0.4 ± 0.04 ^{a}	0.33 ± 0.01 ^{a}	0.34 ± 0.07 ^{a}	1.74 ± 0.09 ^{a}	7.32 ± 0.28 ^{c}	11.96 ± 0.31 ^{c}	1.94 ± 0.16 ^{b}	0.54 ± 0.06 ^{a}
Aerial parts	MeOH	2.24 ± 0.37 ^{b}	0.54 ± 0.05 ^{b}	2.35 ± 0.06 ^{c}	5.32 ± 0.26 ^{b}	2.02 ± 0.04 ^{a}	10.18 ± 0.09 ^{b}	1.04 ± 0.1 ^{a}	0.7 ± 0.05 ^{b}
	Water	2.02 ± 0.38 ^{b}	2.85 ± 0.11 ^{c}	1.66 ± 0.08 ^{b}	5.66 ± 0.39 ^{b}	3.94 ± 0.1 ^{b}	7.14 ± 0.1 ^{a}	1.62 ± 0.21 ^{b}	0.96 ± 0.06 ^{c}
	EA	0.3 ± 0.04	0.17 ± 0.03 ^{A}	1.33 ± 0.09 ^{B}	5.61 ± 0.34 ^{C}	29.07 ± 0.52 ^{B}	21.67 ± 0.63 ^{C}	1.81 ± 0.13 ^{B}	0.97 ± 0.04
Bulbs	MeOH	0.23 ± 0.05	0.24 ± 0.06 ^{B}	1.13 ± 0.09 ^{A}	2.06 ± 0.18 ^{A}	23.05 ± 0.42 ^{A}	14.22 ± 0.35 ^{B}	1.69 ± 0.08 ^{B}	0.92 ± 0.09
	Water	0.27 ± 0.05	0.61 ± 0.02 ^{C}	1.24 ± 0.04 ^{A,B}	2.63 ± 0.11 ^{B}	41.29 ± 0.77 ^{C}	9.56 ± 0.51 ^{A}	1.38 ± 0.04 ^{A}	1.04 ± 0.06

Values are expresses as mean values (mg equivalents/g) ± standard deviation (n=3). Superscript letters within each organ part indicate homogeneous sub-classes as resulted from ANOVA ($p < 0.05$; Duncan's post-hoc test)
