# Supplementary material for: Untargeted Phenolic Profiling and Functional Insights of the Aerial Parts and Bulbs of Drimia maritima (L.) Stearn
Source: Plants (Basel). 2022 Feb 23;11(5):600. doi: 10.3390/plants11050600 (PMC8912325; doi:10.3390/plants11050600)
Supplement: Supplementary file 1 [file plants-11-00600-s001.zip › Table S4 - VIP markes.pdf]

Table S4. Discriminant phenolic compounds selected by using VIP (variable importance in projection) method following supervised OPLS-DA modelling as function of the different extraction solvents. The first block represents the discriminant compounds of aerial parts, while the second block represents those characteristics of bulbs part. Compounds are provided together with VIP scores ± standard error (VIP ±SE) and Log Foldchange values obtained by pair wise comparison among water and ethyl acetate extracts (H2O vs EA), methanol and ethyl acetate extracts (MeOH vs EA), and methanol and water extracts (MeOH vs H2O).

AERIAL PARTS					
**Clsss**	**Primary ID**	**VIP ± SE**	**Log FC A [H2O] vs [EA]**	**Log FC A [MeOH] vs [EA]**	**Log FC A [MetOH] vs [H2O]**
Flavonoids	Pelargonidin	1.25 ± 0.23	-1.51	0.64	2.15
	Cyanidin 3-O-(6''-succinyl-glucoside)	1.24 ± 0.42	-4.00	0.73	4.00
	Petunidin 3-O-(6''-acetyl-glucoside)	1.23 ± 0.43	1.28	2.81	1.54
	Pelargonidin 3,5-O-diglucoside	1.20 ± 0.35	2.61	-0.24	-2.85
	Cyanidin 3-O-(6''-acetyl-glucoside)	1.20 ± 0.38	4.00	0.00	-4.00
	Naringenin	1.22 ± 0.32	0.48	1.98	1.50
	6-Geranylnaringenin	1.23 ± 0.31	-4.00	0.34	4.00
	Apigenin	1.25 ± 0.23	-1.49	0.63	2.12
	Chrysoeriol 7-O-(6''-malonyl-glucoside)	1.24 ± 0.39	0.00	4.00	4.00
	Luteolin 7-O-(2-apiosyl-6-malonyl)-glucoside	1.22 ± 0.35	0.36	-4.00	-4.00
	Isorhamnetin 3-O-glucuronide	1.24 ± 0.34	-4.00	2.52	4.00
	6,8-Dihydroxykaempferol	1.20 ± 0.36	4.00	0.00	-4.00
	Kaempferol 3-O-(2''-rhamnosyl-6''-acetyl-galactoside) 7-O-rhamnoside	1.20 ± 0.34	-4.00	0.64	4.00
	6''-O-Acetylglycitin	1.22 ± 0.37	1.16	-4.00	-4.00
Lignans	Arctigenin	1.24 ± 0.31	4.00	4.00	4.92
	Lariciresinol-sesquilignan	1.23 ± 0.41	4.66	5.95	1.29
	Medioresinol	1.2 ± 0.34	1.66	-0.20	-1.86
Other polyphenols	5-Heneicosylresorcinol	1.24 ± 0.26	-4.00	1.15	4.00
	Carnosol	1.24 ± 0.38	-4.00	4.18	4.00
	p-HPEA-AC	1.24 ± 0.30	0.82	-4.00	-4.00
	Oleuropein-aglycone	1.23 ± 0.38	2.22	3.59	1.37

	3,4-DHPEA-EDA	1.22 ± 0.35	1.53	-2.55	-4.09
Phenolic acids	Protocatechuic acid 4-O-glucoside	1.21 ± 0.35	-2.61	0.06	2.67
	Rosmarinic acid	1.24 ± 0.25	-3.12	0.94	4.06
	Chicoric acid	1.20 ± 0.42	2.57	-0.68	-3.25

BULBS

**Class**	**Primary ID**	**VIP ± SE**	**Log FC R [H2O] vs [EA]**	**Log FC R [MetOH] vs [EA]**	**Log FC R [MetOH] vs [H2O]**
Flavonoids	Cyanidin 3-O-glucoside	1.25 ± 0.29	-1.03	1.63	2.66
	Pelargonidin	1.24 ± 0.36	1.89	-1.14	-3.03
	Cyanidin 3-O-glucosyl-rutinoside	1.24 ± 0.34	0.33	-0.96	-1.29
	Vitisin A	1.23 ± 0.37	3.49	-4.00	-4.00
	Cyanidin 3-O-(6''-malonyl-glucoside)	1.23 ± 0.37	4.00		-4.00
	Petunidin 3,5-O-diglucoside	1.23 ± 0.35	0.80	2.82	2.02
	Pelargonidin 3-O-glucoside	1.22 ± 0.36	-0.94	0.98	1.91
	(+)-Catechin 3-O-glucose	1.23 ± 0.35	0.09	5.86	5.77
	Dihydroquercetin	1.23 ± 0.37	-1.06	0.75	1.82
	Theaflavin	1.25 ± 0.28	-2.19	2.20	4.39
	Narirutin 4'-O-glucoside	1.23 ± 0.37	3.65	-0.36	-4.01
	Luteolin 6-C-glucoside	1.25 ± 0.29	-0.98	1.60	2.58
	Apigenin 6,8-di-C-glucoside	1.23 ± 0.35	0.39	1.66	1.27
	Apigenin 6-C-glucoside	1.22 ± 0.36	-0.90	0.97	1.87
	Isorhoifolin	1.20 ± 0.46	-0.48	0.57	1.05
	Jaceidin 4'-O-glucuronide	1.24 ± 0.35	1.86	-4.00	-4.00
	Quercetin 3-O-(6''''-malonyl-glucoside)	1.24 ± 0.38	2.43	-4.00	-4.00
	Quercetin 3-O-(6''-acetyl-galactoside) 7-O-rhamnoside	1.23 ± 0.37	4.00		-4.00
	Quercetin 3-O-glucosyl-rhamnosyl-galactoside	1.21 ± 0.45	4.00		-4.00
	Kaempferide	1.20 ± 0.50	3.47	0.58	-2.89
	Quercetin 7,4'-O-diglucoside	1.20 ± 0.39	4.00	4.00	2.54
Lignans	Secoisolariciresinol-sesquilignan	1.24 ± 0.32	0.53	-3.18	-3.71
	Medioresinol	1.21 ± 0.41	3.01	1.31	-1.70
Other polyphenols	5-Heptadecylresorcinol	1.22 ± 0.37	5.26	2.04	-3.21
	Psoralen	1.23 ± 0.37	3.26	-4.00	-4.00
	Esculin	1.22 ± 0.36	3.59	1.29	-2.29

	p-HPEA-AC	1.22 ± 0.35	4.20	1.89	-2.31
Phenolic acids	3,4-Dicaffeoylquinic acid	1.23 ± 0.36	2.43	-4.00	-4.00
	p-Coumaroyl tartaric acid	1.21 ± 0.41	0.41	2.30	1.89
Stilbenes	Resveratrol	1.22 ± 0.43	-1.22	0.53	1.75
